# Knowledge, Attitude and Practice Toward COVID-19: A Cross-Sectional Study of Staff in China-Guinea Friendship Hospital, Guinea

**DOI:** 10.3389/fpubh.2022.889227

**Published:** 2022-05-30

**Authors:** Wenyan Ji, M'Bemba Abdoulaye Camara, Jinggang Xia, Xiaobo Ma, Xintong Chen, Ying Liu

**Affiliations:** ^1^Beijing Center for Disease Prevention and Control, Beijing Research Center for Preventive Medicine, Institute for Immunization and Prevention, Beijing, China; ^2^Department of Emergency, China-Guinea Friendship Hospital, Conakry, Guinea; ^3^Department of Cardiology, Xuanwu Hospital, Capital Medical University, Beijing, China; ^4^Department of Finance, Xuanwu Hospital, Capital Medical University, Beijing, China; ^5^Department of Pharmacy, Xuanwu Hospital, Capital Medical University, Beijing, China; ^6^Office of Medical Affairs, Beijing Municipal Heath Commission, Beijing, China

**Keywords:** COVID-19, hospital staff, knowledge, attitude, practice, Guinea (Conakry)

## Abstract

**Background:**

The purpose of this study was to assess the level of knowledge, attitude and practice of COVID-19 among staff in China-Guinea Friendship Hospital, and to confirm the effect of nosocomial infection management.

**Methods:**

This cross-sectional study was conducted in December 2021. Information on socio demographic data, knowledge, attitude and practices related to COVID-19 was collected through a self-administered questionnaire.

**Results:**

A total of 143 employees participated in the survey, with a response rate of 99.31% and a vaccination rate of 95.10%. The average knowledge score of COVID-19 was 8.39 ± 1.3 points (10 points in total), without significant differences between subgroups with different demographic variables (*P* > 0.05); more than 80% of the participants had a positive attitude, and 72.03–93.01% of the participants could take appropriate preventive practices in different environments such as hospital, outdoor or home.

**Conclusion:**

The staff of the China-Guinea Friendship Hospital has good knowledge of COVID-19, a positive attitude and appropriate preventive practices. It can be concluded that the current nosocomial infection management is active and effective. Therefore, this study suggests that comprehensive activities such as training, promotion and supervision of COVID-19-related knowledge and countermeasures should be widely and continuously implemented in healthcare facilities, which will continuously improve the overall KAP level of hospital staff and play an important role in curbing the COVID-19 pandemic.

## Introduction

Coronavirus disease 2019 (COVID-19) is a novel highly contagious respiratory disease caused by a novel coronavirus. It was first detected in Wuhan, China in December 2019. World Health Organization (WHO) declared COVID-19 was a global pandemic disease on March 11th, 2020 (1). As of February 25th, 2022, over 432 million confirmed cases and about 6 million deaths have been reported globally. According to the Guinean Ministry of Health and Security, the first case was confirmed on March 14th, 2020. Guinea reported 36,393 confirmed cases and 440 deaths on March 25th, 2022 (2).

People's knowledge, attitude and practice (KAP) toward COVID-19 are fairly crucial and critical to ensuring successful disease control (3, 4). In the KAP assessment of COVID-19, knowledge usually refers to the level of mastery of biomedical concepts (5). Typical questions for knowledge assessment include causes and symptoms of COVID-19. Attitude is expressed in people's beliefs, emotions and tendencies. Practice refers to the lifestyle related to preventing COVID-19.KAP studies provide baseline information for identifying interventions (6), and can be used to assess the appropriateness of existing interventions (7). Hospital staff, who are on the front lines of the COVID-19 pandemic, are more vulnerable to infection. If they have insufficient knowledge about COVID-19 and/or inappropriate preventive behavior, treatment will be delayed and COVID-19 will spread rapidly (8, 9). Therefore, hospital staff have been an important population for assessing the KAP of COVID-19. In addition to relying on information and resources from WHO and governments, appropriate nosocomial infection management also plays an important role in improving KAP levels in healthcare workers (10).

The China-Guinea Friendship Hospital is located in Conakry, the capital of Guinea, and is one of the many hospitals jointly built by China and African countries. In March 2021, the 28th Chinese Medical Aid Team to Guinea and the hospital jointly established the Nosocomial Infection Management Committee, and subsequently carried out a series of nosocomial infection prevention and control work. To assess the effects of these interventions and provide a basis for adjusting interventions, a KAP questionnaire survey toward COVID-19 among hospital staff was conducted in December 2021.

## Materials and Methods

### Study Design and Subjects

This cross-sectional study was conducted among all staff at the China-Guinea Friendship Hospital in Conakry, Guinea, during December 2021. A self-administered questionnaire was used to assess subjects' KAP levels related to COVID-19. Participants gave informed consent to be included in the study, and the study protocol was approved by the ethics committee of China-Guinea Friendship Hospital.

Those employees who were working at the hospital at that time and had a direct employment relationship with the hospital were included in the study, with no exceptions.

### Questionnaire

The questionnaire consisted of four parts. The first part included questions about the demographic characteristics of the participants (age, gender, the specific department, working years, specific job categories and whether vaccinations were administered, etc.). The other three parts in the questionnaire were COVID-19-related KAP questions. There were 10 questions in each part (For the knowledge part, each question was assigned 1 point, a total of 10 points). The questions of knowledge included the etiology, epidemiology, pathogenesis of COVID-19. The information collected by the attitude questions included: the degree of concern and worry about the epidemic, the degree of concern for one's own health, the confidence in curbing its spread, the satisfaction with the cleaning, disinfection and material supply of the hospital, as well as the satisfaction of training and information exchange of the hospital, etc. The questions of practice included the participants' self-protection in different scenarios, such as outdoors, workplace and home.

For the convenience of participants, the questionnaire was in French.

### Statistical Analysis

Descriptive statistics were used to present demographic data, participants' knowledge scores, and the frequency of COVID-19 knowledge, attitude and preventive practice. The 95% confidence intervals (95% CI) were compared with or without crossover to determine the differences in values between subgroups with different characteristics. For continuous variable (such as age, working years), if it is a normal distribution, it is divided into two groups by the mean; if it is a skewed distribution, it is divided into two groups by the median; the specific departments were grouped into three big sectors: medical sector, medical technology sector, administrative and logistic sector; in addition, the specific positions were divided into medical-related positions and non-medical-related positions. Data were coded and analyzed by SAS software (version 19.0; SAS Institute Inc., Cary, NC).

## Results

### Participants' General Characteristics

A total of 143 participants completed the questionnaire with a response rate of 99.31% (143/144), except for one logistics staff member, who was unable to participate due to intellectual problems. Of all respondents, 58.74% (84/143) were males, 75.52% (108/143) were in medical-related positions, with a mean age of 42.86 ± 11.60 years, ranging from 22 to 65 years. Regarding the sector of work, 65.03% (93/143) were in the medical sector, 17.48% (25/143) in the medical technology sector, and 17.48% (25/143) in the administrative and logistic sector. The range of participants' working years was 1–39 years, with a Quartile (P25, P75) of 9 (4, 16) years. And the vaccination rate of participants was 95.10% (136/143).

### Participants' Knowledge Toward COVID-19

The overall accuracy rate was 83.92% for the knowledge. More than 90% knew the main sources of COVID-19 transmission, the main symptoms, the incubation period of the disease, the effectiveness of the vaccine, and the role of chlorine-containing disinfectants. The proportion of people who correctly understood the knowledge of the transmission route, infectivity, susceptible population, and the presence of seasonal patterns of the virus varied between 54.55 and 79.72% (Table 1).

**Table 1:** Participants' correct responses to questions bordering on knowledge of COVD-19 (*n* = 143).

**Knowledge items**	***N***	**Proportion (%)**
1. Infected persons (patients and asymptomatic infected persons) are the main source of infection	135	94.41
2. Droplet transmission is its main mode of transmission	114	79.72
3. The virus is not transmitted by aerosols or dirt	109	76.22
4. At the end of the incubation period is infectious, and the infection is relatively strong in the first 2 days of the disease.	109	76.22
5. The elderly and children are not susceptible to COVID-19	114	79.72
6. Some patients have fever, dry cough and weakness as the main symptoms, some patients have loss of smell and taste as the first symptoms, a few patients have nasal congestion, runny nose, sore throat, conjunctivitis, myalgia and diarrhea, etc.	138	96.50
7. The incubation period of COVID-19 virus infection is usually 1-14 days, mostly 3-7 days.	132	92.31
8. COVID-19 epidemic has a significant seasonality.	78	54.55
9. Vaccination can reduce morbidity.	138	96.50
10. The virus is sensitive to chlorine-containing disinfectants.	133	93.01
**Mean**	**120**	**83.92**

The COVID-19 knowledge scores in this study were normally distributed, with an average of 8.39 ± 1.3 points. At the level of α = 0.05, there were no significant differences in which between subgroups with different characteristics (including department, gender, age, years of work, and whether the position was related to medicine).

### Participants' Attitude Toward COVID-19

Participants' attitude toward COVID-19 is shown in Table 2. Of all the respondents, 99.30% expressed “concern” and “worry” about the pandemic; 100% were “concerned” about their own health during the pandemic; 65.03% expressed “confidence” that the pandemic would be overcome in the end; 92.31% and 86.71% were “particularly satisfied” with the current environmental cleanliness and disinfection of the hospital, respectively; 89.51% thought that the current quantity and quality of the hospital's supply and stock of epidemic prevention materials could “meet the needs”; 74.83% were “particularly satisfied” with the hospital's training in knowledge and techniques of COVID-19; and 84.62% were “particularly satisfied” with the hospital's current communication and delivery of information.

**Table 2:** Responses of the participants to the attitude items on the questionnaire (*n* = 143).

**Attitude items**	**Categories**	***N***	**Proportion (%)**
1. Degree of concern in information toward the COVID-19:	Particularly concern	129	90.21
	Concern	13	9.09
	No concern	1	0.70
2. Degree of worry in information toward the COVID-19:	Particularly worry	118	82.52
	Worry	24	16.78
	No worry	1	0.70
3. Degree of concern your own health during the pandemic:	Particularly concern	126	88.11
	concern	17	11.89
	No concern	0	0.00
4. Confidence in the ability to overcome the COVID-19:	Have confidence	93	65.03
	Not easy to say	47	32.87
	No confidence	2	1.40
	Doesn't matter	1	0.70
5. Degree of satisfaction with the current environmental cleanliness in the hospital:	Particularly satisfy	132	92.31
	Not sure	1	0.70
	No satisfy	9	6.29
	Doesn't matter	1	0.70
6. Degree of satisfaction with the current environmental disinfection in the hospital	Particularly satisfy	124	86.71
	Not sure	10	6.99
	No satisfy	8	5.59
	Doesn't matter	1	0.70
7. In terms of the number, the extent to which the current supply and stockpile of epidemic prevention materials in the hospital meet the needs of the post:	Particularly sufficient	49	34.27
	Tightly meet the needs only	79	55.24
	Can't meet the needs	9	6.29
	Not sure	6	4.20
8. In terms of the type, the extent to which the current supply and stockpile of epidemic prevention materials in the hospital meet the needs of the post:	Particularly sufficient	50	34.97
	Tightly meet the needs only	78	54.55
	Can't meet the needs	8	5.59
	Not sure	7	4.90
9. Degree of satisfaction with relevant knowledge and technical training within the hospital:	Particularly satisfy	107	74.83
	Not sure	10	6.99
	No satisfy	15	10.49
	Doesn't matter	11	7.69
10. Degree of satisfaction with the communication and delivery of information related to the epidemic in the hospital:	Particularly satisfy	121	84.62
	Not sure	8	5.59
	No satisfy	6	4.20
	Doesn't matter	8	5.59

In addition, 35.97% expressed “not easy to say” or “no confidence” or “Doesn't matter” in overcoming the epidemic, and they were widely distributed across departments, with the top three departments being neurosurgery, abdominal surgery and emergency department.

### Participants' Practice Toward COVID-19

The results of participant's practices toward COVID-19 are shown in Table 3. The proportion of wearing masks in public places was 93.01%; 73.43% intentionally reduced unnecessary outings (such as parties, meals, etc.); 72.03% of people were careful to maintain a social distance of at least one meter; 96.5% washed their hands ≥3 times a day; 46.15% opened windows ≥2 times a day; 79.72% disinfected the environment and objects; 43.36% strengthened physical exercise; 82.52% carried out garbage sorting; and 89.51% of staff wore disposable medical masks at work. When there were symptoms of suspected infection such as fever, fatigue, and dry cough, 97.90% of them chose to seek medical treatment.

**Table 3:** Responses of the participants to the practice items on the questionnaire (*n* = 143).

**Attitude items**	**Categories**	***N***	**Proportion (%)**
1. Do you wear a mask when you are in public places during an epidemic?	Always	133	93.01
	Occasionally	1	0.70
	Never	0	0.00
2. Do you intentionally reduce on unnecessary outings (e.g., fewer parties, meals, etc.) during the epidemic?	Always	105	73.43
	Occasionally	35	24.48
	Never	3	2.10
3. Are you careful to maintain a social distance of at least one meter during the epidemic?	Always	103	72.03
	Occasionally	37	25.87
	Never	3	2.10
4. During the epidemic, how many times a day do you wash your hands?	<3 times/day	5	3.50
	≥. times/day	13	9.09
	≥7 times/day	33	23.08
	≥10 times/day	92	64.34
5. During the epidemic, how many times do you open the windows in your room (office or home) to ventilate?	0 time/day	38	26.57
	1 time/day	39	27.27
	2 times/day	22	15.38
	≥3 times/day	44	30.77
6. During the epidemic, do you pay attention to the disinfection of the environment and goods?	Always	114	79.72
	Occasionally	27	18.88
	Never	2	1.40
7. During the epidemic, do you intend to be more physically active?	Always	62	43.36
	Occasionally	67	46.85
	Never	14	9.79
8. Do you sort your garbage?	Always	118	82.52
	Occasionally	25	17.48
	Never	0	0.00
9. What do you do when you feel fever, malaise, dry cough and other suspected symptoms of infection during an outbreak? (Multiple choice possible)	Seeking Medical Attention	140	97.90
	Home isolation	29	20.28
	Go to work normally	2	1.40
	Concealment of illness and refusal to seek medical attention	1	0.70
10. During the epidemic, what is your mode of protection during work? (Multiple choice possible)	Wearing disposable caps	107	74.83
	Wearing disposable medical masks	128	89.51
	Wearing disposable non-medical masks	24	16.78
	Wearing disposable gloves	113	79.02
	Wear goggles and face screen	85	59.44
	Wear a disposable barrier suit	91	63.64
	Wear disposable protective clothing	63	44.06
	No protective measures	2	1.40
	Other	0	0.00

## Discussion

Today, every country in the world is facing the COVID-19 pandemic. At present, taking preventive measures is the only effective way to copy with this infectious disease for which there is no effective treatment. The preventive effect is largely dependent on the KAP level of susceptible populations, and particularly, the KAP level of hospital staff is more important in controlling the spread of COVID-19 (10). Since the establishment of the nosocomial Infection Management Committee of the China-Guinea Friendship Hospital in March 2021, a series of COVID-19 prevention and control measures have been carried out in the hospital, including training in the knowledge and techniques of COVID-19, developing a prevention and control system, strengthening supervision and inspection, and replenishing epidemic prevention materials through multiple channels. The results of this study showed that most staff had good knowledge, positive attitude and appropriate preventive practices in the prevention and control of COVID-19, which identify that nosocomial infection management measures are active and effective.

The results of this study showed that the staff of the China-Guinea Friendship Hospital had a good knowledge of COVID-19, with an overall correct rate of 83.92%. This result is higher than that of surveys in the general population (11, 12) and some hospital workers (10, 13), whose overall correct knowledge estimates ranged from 48.97 to 77.00%. There are also some studies conducted among hospital staff (whose overall correct rate of knowledge was estimated to be 80–90%) consistent with our results (14–17). Of course, there are also some studies of hospital workers that had higher results than ours, up to 90% or more (18–21). It is worth noting that in addition to the different survey populations, the results of each study may also vary due to other factors, such as knowledge definition standards, question design, survey methods, and the development stage of the epidemic at the time of the survey.

In this study, although hospital staff had a level of knowledge above 90% on the source of virus transmission, main clinical symptoms, incubation period, vaccines and the effects of chlorinated disinfectants, the level of knowledge on other issues remained low, such as virus transmission route, virus infectivity, susceptible population and whether there is seasonality, etc., their correct rate of was 53.96–79.86%. Because more scientific knowledge is gradually enriched and proposed with the progress of the epidemic, we need to continuously enrich and update the relevant training for hospital staff and strengthen weak knowledge points.

In terms of attitude, more than 80% of the staff of China-Guinea Friendship Hospital had a positive attitude. This result is higher than those of previous studies among medical staff (those participants had a moderate or positive attitude rate of 50.5–72.2%) (10, 22–24), and the reason may be related to the higher rate of knowledge correctness (17, 23, 25). Almost 65.03% of hospital staff believed that COVID-19 would eventually be overcome in our study, which is similar to the results of some previous studies (10). The staff of low-confidence in this study were mainly concentrated in the emergency, neurosurgery and abdominal surgery. There are two possible reasons: on the one hand, there are a large number of staff in these departments; on the other hand, as the front line of the hospital's prevention and control, these staff are under great mental pressure. Therefore, it is necessary to strengthen training, especially encourage and support these important departments, so as to enhance their confidence in overcoming the epidemic.

In the term of practice, ~72.03–93.01% of staff had appropriate protective practices at different places, such as workplace, outside or at home. The results are better than the results of a systematic review and meta-analysis, which researched on globally practice studies of COVID-19 by 70% [95% CI (66, 74%)], with Africa practice score lower than 60% (11). Effective preventive measures, such as swearing masks, hand hygiene, vaccinations, and maintaining safe social distancing, can reduce the transmission of COVID-19, which is always recommended by World Health Organization. And people's adherence to preventive measures is affected by their COVID-19 knowledge and attitude (25–27).

Based on the survey results, it is recommended to continue to strengthen and enrich knowledge training associated with COVID-19 in healthcare facilities, strengthen inspection, supervision, encouragement and support focusing on front-line departments to protect the health of hospital staff and patients, which will play an important role in curbing the COVID-19 pandemic.

## Limitations

The major limitation of this study is the lack of the control, resulting in a weak persuasive power. One more methodological limitation is that the questionnaire was self-administered by the respondents and was not based on objective observations, which resulted in a certain degree of information bias.

## Data Availability Statement

The raw data supporting the conclusions of this article will be made available by the authors, without undue reservation.

## Ethics Statement

The studies involving human participants were reviewed and approved by the Ethics Committee of China-Guinea. The patients/participants provided their written informed consent to participate in this study.

## Author Contributions

All authors made substantial contributions to conception and design, acquisition of data, or analysis and interpretation of data, took part in drafting the article or revising it critically for important intellectual content, gave final approval of the version to be published, and agreed to be accountable for all aspects of the work.

## Conflict of Interest

The authors declare that the research was conducted in the absence of any commercial or financial relationships that could be construed as a potential conflict of interest.

## Publisher's Note

All claims expressed in this article are solely those of the authors and do not necessarily represent those of their affiliated organizations, or those of the publisher, the editors and the reviewers. Any product that may be evaluated in this article, or claim that may be made by its manufacturer, is not guaranteed or endorsed by the publisher.
